# A 2D–3D hybrid convolutional neural network for lung lobe auto-segmentation on standard slice thickness computed tomography of patients receiving radiotherapy

**DOI:** 10.1186/s12938-021-00932-1

**Published:** 2021-09-23

**Authors:** Hengle Gu, Wutian Gan, Chenchen Zhang, Aihui Feng, Hao Wang, Ying Huang, Hua Chen, Yan Shao, Yanhua Duan, Zhiyong Xu

**Affiliations:** Shanghai Chest Hospital, Shanghai Jiao Tong University, Shanghai, China

**Keywords:** Artificial intelligence, Computed tomography, Automatic segmentation, Lung lobe, Convolutional neural network

## Abstract

**Background:**

Accurate segmentation of lung lobe on routine computed tomography (CT) images of locally advanced stage lung cancer patients undergoing radiotherapy can help radiation oncologists to implement lobar-level treatment planning, dose assessment and efficacy prediction. We aim to establish a novel 2D–3D hybrid convolutional neural network (CNN) to provide reliable lung lobe auto-segmentation results in the clinical setting.

**Methods:**

We retrospectively collected and evaluated thorax CT scans of 105 locally advanced non-small-cell lung cancer (NSCLC) patients treated at our institution from June 2019 to August 2020. The CT images were acquired with 5 mm slice thickness. Two CNNs were used for lung lobe segmentation, a 3D CNN for extracting 3D contextual information and a 2D CNN for extracting texture information. Contouring quality was evaluated using six quantitative metrics and visual evaluation was performed to assess the clinical acceptability.

**Results:**

For the 35 cases in the test group, Dice Similarity Coefficient (DSC) of all lung lobes contours exceeded 0.75, which met the pass criteria of the segmentation result. Our model achieved high performances with DSC as high as 0.9579, 0.9479, 0.9507, 0.9484, and 0.9003 for left upper lobe (LUL), left lower lobe (LLL), right upper lobe (RUL), right lower lobe (RLL), and right middle lobe (RML), respectively. The proposed model resulted in accuracy, sensitivity, and specificity of 99.57, 98.23, 99.65 for LUL; 99.6, 96.14, 99.76 for LLL; 99.67, 96.13, 99.81 for RUL; 99.72, 92.38, 99.83 for RML; 99.58, 96.03, 99.78 for RLL, respectively. Clinician's visual assessment showed that 164/175 lobe contours met the requirements for clinical use, only 11 contours need manual correction.

**Conclusions:**

Our 2D–3D hybrid CNN model achieved accurate automatic segmentation of lung lobes on conventional slice-thickness CT of locally advanced lung cancer patients, and has good clinical practicability.

## Background

Lung cancer is one of the most devastating tumors with a high incidence and mortality worldwide [1]. Radiotherapy is considered the main option for locally advanced lung cancer patients. Although radiotherapy improves locoregional control and survival in patients with lung cancer, radiation-induced lung injury (RILI) is common treatment-related toxicity, which can be fatal in severe cases. Due to the large tumor volume and extensive lymph node involvement, patients at locally advanced stage would risk inadequate dose delivery to the target because of dose limitations arising from adjacent critical organs. Some of them may even lose the opportunity to receive curative treatment. Intensity-modulated radiotherapy produces accurate dose homogeneity around targets and less toxicity to normal organs by optimizing 3D dose distributions based on dose constraints prescribed for the target and normal tissues. In previous studies, most of the dosimetric constraints used standardly refer to both lungs as a single functional unit [2–6]. Some recent studies [7–9] suggest that lobar level treatment planning and radiation dose assessment may be an accessible way to improve treatment planning and reduce the incidence of radiation-induced lung injury. Radiation oncologists often need to manually delineate the tumor and normal tissues slice-by-slice on CT images acquired for radiotherapy planning. Manual segmentation of the lung lobe is time-consuming and has poor replicability among different observers. Therefore, there is the prerequisite to develop a fully automatic methodology that produces reliable segmentation of the lung lobe in the clinical setting.

**Fig. 1:**
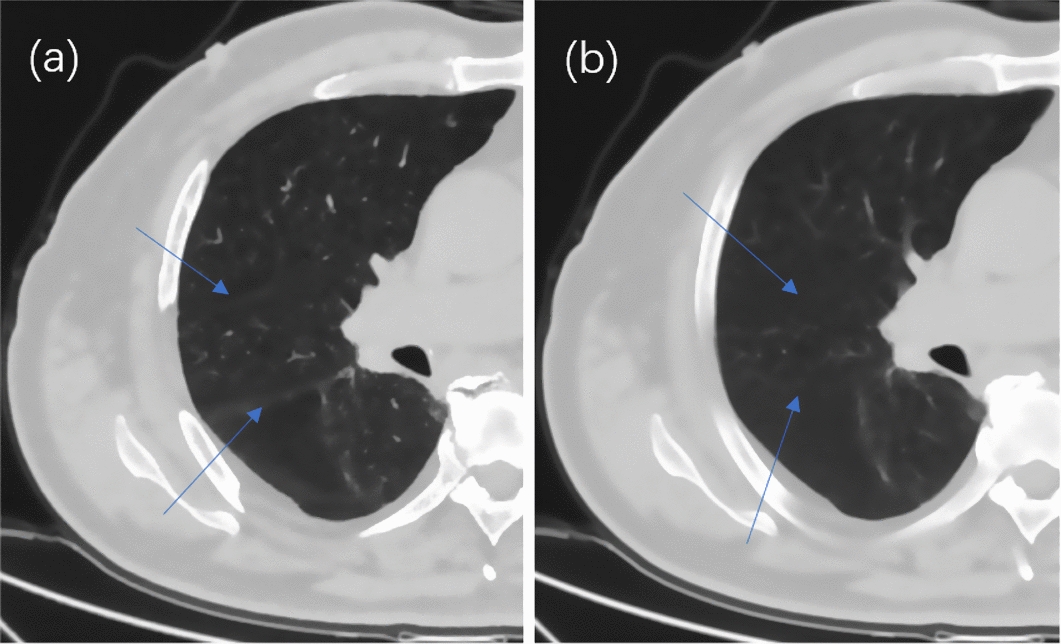
Axial view of right lung for one patient. Blue arrows point to the fissures in right lung. **a** 1 mm slice CT, **b** 5 mm slice CT

For locally advanced lung cancer patients undergoing radiotherapy, the treatment planning CT is usually acquired with 5 mm slice thickness, this adds to the difficulty of lung lobe auto-contouring, since slice thickness will generally affect the tissue contours in the images. The lungs have five different partitions called lobes. The left lung is divided into upper and lower lobes, and the right lung is divided into upper, middle, and lower lobes [10]. The boundaries of each lobe are fissures. The thin-section CT was beneficial for recognizing the fissures. As shown in Fig. 1, it was clear that the interlobar fissures often appear as a cavity without the intersection of the vascular tree or the bronchial tree on a standard CT, while they are displayed as lines on thin-section CT [11]. Most of the existing technologies rely on pulmonary blood vessel/airway segmentation or semi-automated methods [12]. However, the segmentation of lung airways and blood vessels is relatively complicated and not always reliable, especially in the presence of disease.

In recent years, in the field of image segmentation, various deep learning-based segmentation algorithms based on 2D or 3D convolutional neural network (CNN) have been proposed. Some studies [13–16] reported the use of deep learning in lung lobe segmentation. George et al. [14] employ a 2D fully convolutional network combined with a 3D random walker refinement. This approach achieves high accuracy without reliance on prior segmentation of the airway or vessel. However, it relied on prior segmentation of the lobar boundaries, and the 3D random walker algorithm needs the initialization of seeds and weights. Harrison et al. [13] adapt the holistically nested network (HNN) with a progressive constraint on multi-scale pathways to overcome issues with HNN output ambiguity and the coarsening resolution of fully convolutional networks. Imran et al. [15] introduced a fast and fully automated lung lobe segmentation method based on a Progressive Dense V-Network. This method can segment lung lobes in one forward pass of the network. Park et al. [16] used a lung lobe segmentation method with 3D U-Net architecture and achieved high accuracy of lobe segmentation on the chest CT scans of mild-to-moderate COPD patients. These studies provide a very important base for further researches. Training a deep neural network that can generalize well to new data is a challenging problem. These reported automatic segmentation algorithms are usually developed and tested on carefully selected high-resolution public data sets, slice thickness ranged from 0.50 to 1.50 mm. However, experiments on conventional imaging data show that algorithms that perform well on public data sets cannot produce accurate and reliable segmentation in clinical CT images of patients with severe diseases [17].

Unlike CT images for diagnostic purposes, radiotherapy planning CT, as the basis for treatment planning, needs to consider the efficiency of treatment and the accuracy of target delineation. There are usually standard slice thicknesses of CT scans for tumors of different volumes at different sites. When patients with locally advanced non-small-cell lung cancer are treated with radiation therapy, a 5-mm slice thickness is generally used to obtain CT images for radiation therapy planning. The increased slice thickness of clinical standard CT affects the image quality, which results in segmentation accuracy reduction. To the best of our knowledge, lung lobe auto-contouring by deep learning method in 5 mm slice CT of lung cancer patients has not been previously investigated.

When it comes to these conventional but not perfect clinical data, it is necessary to make modifications to the learning model network architecture such that improves the model’s performance on these data. The 2D network is inefficient and cannot capture inter-slice correlations. To learn volumetric information in CT images, the convolution kernels is to extend from 2 to 3D. In this way, the networks can take full advantage of the 3D context for better performance. But 3D CNN has more parameters than 2D CNN, and the training of 3D CNN is computationally expensive, which limits the construction of very deep networks. In 5 mm slice CT images, the voxel scale in the Z-axis is much larger than that in the XY plane. Directly performing 3D convolutions with isotropic kernels on these anisotropic volumetric images could be problematic.

In this study, we propose a 2D–3D hybrid segmentation network based on a convolutional neural network to automatically segment lung lobes from 5 mm slice-thickness computed tomography (CT) images. The proposed network combines the advantages of both 2D and 3D CNN, improving the model accuracy, and achieved high performances in this challenging data set. Its purpose is to assist clinicians in their decision-making with lobar level treatment planning, radiation dose assessment, and efficacy prediction, which may help to improve treatment outcomes and reduce toxicity for locally advanced lung cancer patients undergoing radiotherapy.

## Results

The tumors of 70 patients in the training & validation set and 35 patients in the test set were distributed in 5 lobes. The tumor location information of all cases is shown in Table 1.

For the 35 patients in the test group, the DSC of the auto-contour of all lobes was over 0.75, which met the pass standard of segmentation result.

**Fig. 2:**
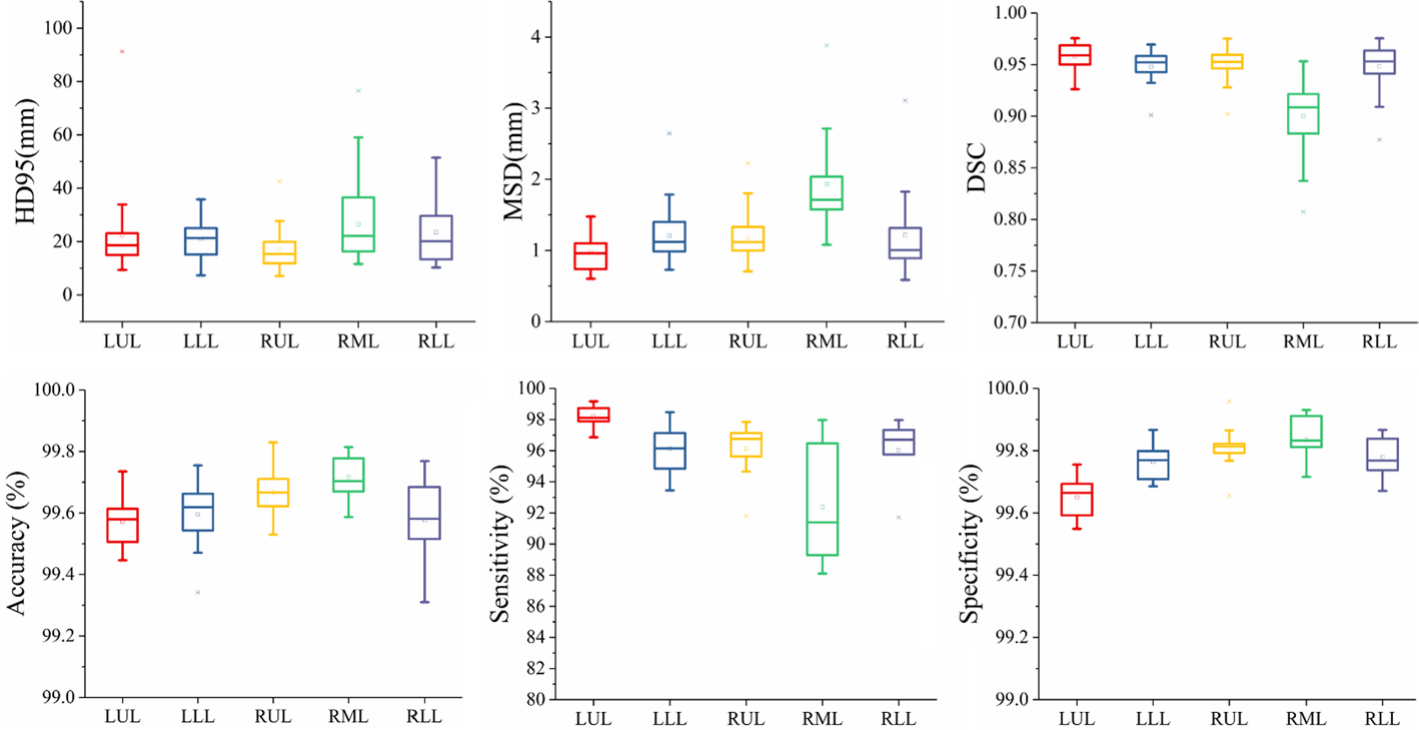
Boxplot of HD95, MSD, DSC, accuracy, sensitivity and specificity

All the automatic segmentation profiles were divided into five groups according to the left upper lobe, left lower lobe, right upper lobe, right middle lobe, and right lower lobe. Six different quantitative indexes, HD95, MSD, DSC, Sensitivity, Specificity, and Accuracy were used for evaluation. Figure 2 shows that DSC results of all the other lobes were around 0.95 except for the mean DSC of the right middle lobe of 0.9003 ± 0.0331. Quantitative parameters for lung lobe contouring are shown in Table 2.

**Fig. 3:**
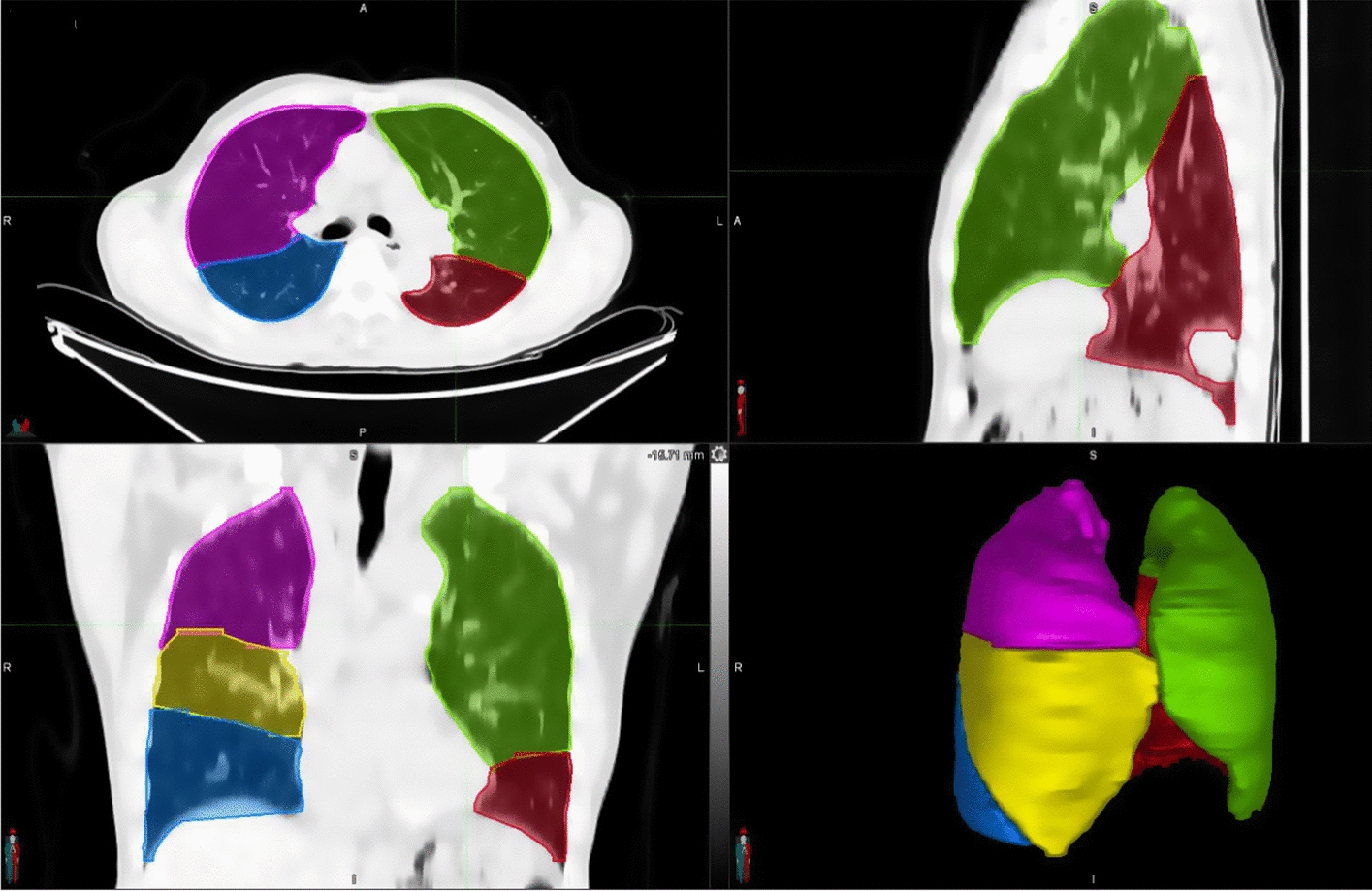
Example of lung lobe contours from our 2D–3D segmentation network

Figure 3 shows an example of lung lobe contours from our 2D–3D hybrid auto-segmentation network. A radiation oncologist performed quality evaluation (accepted as is/need manual correction/failed) on the segmentation results of our lung lobe segmentation model on 35 cases in the test group in slice (axial, coronal, sagittal) and 3Dviews. Among the 175 lung lobe contours from 35 cases, 164 (93.7%) were accepted as is; only 11 (6.2%) of the lung lobes, distributed in a total of 28 slices, need manual correction before clinical use; 0(0%) failed.

**Fig. 4:**
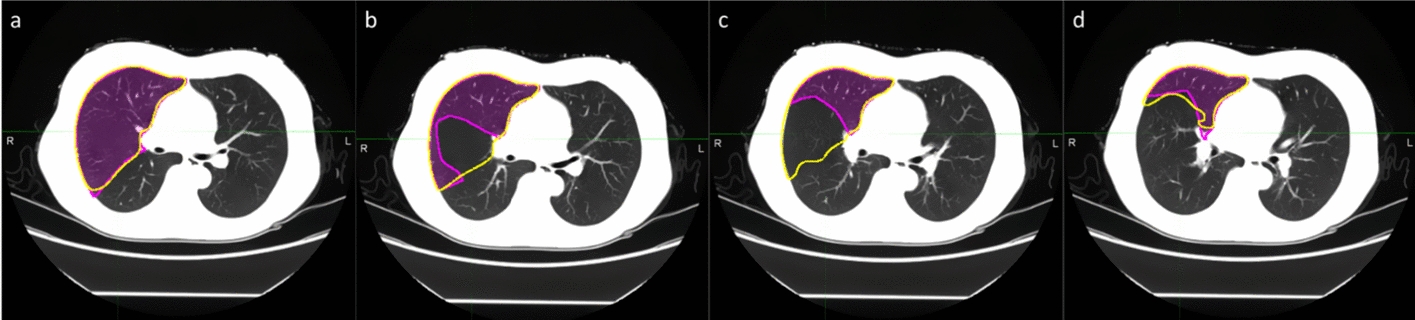
Example of auto-segmentation requires manual correction

Figure 4 shows an example case in which there are differences between the automatical segmentation and the manual contour of RUL. Figure 4a–d shows four consecutive slices in the same CT sequence. Due to the sudden occurrence of fissures, the right upper lobe contours vary greatly between consecutive slices, and the automatic segmentation network failed to accurately identify these changes until the contour of the right middle lobe appears in slice (d).

## Discussion

In this study, we proposed a 2D–3D hybrid CNN model for lung lobes segmentation on the conventional 5 mm slice-thickness CT images of locally advanced NSCLC patients. The segmentation results were assessed using quantitative indexes, all contours passed the criteria with DSC >  = 0.75. Our segmentation results have been visually evaluated by the clinician. Most of the segmentation results can be clinically acceptable as is except for a few (11 / 175 contours) which need minor manual correction. Our model greatly alleviates the workload of the clinician in manually contouring the lung lobes and provides help for lobar-level contouring, treatment planning, dose distribution prediction, and evaluation on 5 mm slice-thickness CT images of locally advanced lung cancer patients.

Before locally advanced NSCLC patients undergoing radiotherapy, planning CT images were obtained on the simulation CT, target and OARs were delineated on the planning CTs, and then the CT images and RT structures were used for treatment planning. Since the treatment volume of these patients includes tumor and lymph nodes, and the irradiation volume is large, their planning CT images are usually acquired with a thickness of 5 mm. Compared with other CT images with thinner thickness, due to the partial volume effect (PVE) in Z-axis, the resolution and contrast of CT images will be reduced, and the thin front and back connecting lines in the images will be affected [18]. In addition, the existence of a tumor in the lung will also damage the structure of the adjacent lung lobe, which increases the difficulty of automatic segmentation. Although there are some commercial automatic contouring softwares provide auto-segmentation of some normal tissues on the radiotherapy planning CT images, but, as far as we know, there is no commercial software that can automatically contour the lung lobes to meet the needs of clinical applications on this type of CT images.

In recent years, some studies [13, 15, 16, 19] have reported the application of deep learning models based on convolutional neural networks in lung lobes segmentation, and many have obtained satisfactory results. Most of the models in these studies use public data sets for model training and verification. Taking into account the difficulties in the automatic segmentation of lung lobes in conventional radiotherapy planning CT images for patients with locally advanced NSCLC, these algorithms that perform well on public data sets cannot produce accurate and reliable segmentation when directly applied to clinical standard images, so we tried to establish a new automatic segmentation model that can meet this clinical need.

The 2D–3D CNN hybrid segmentation model we proposed uses 3D CNN to learn 3D volumetric information [14] while taking advantage of 2D CNN to extract the edge information within the layers [13] to maximize the potential of the algorithm on limited data. Thereby improving the accuracy of segmentation. Our test results on a test set containing 35 cases and 175 segmentation contours show that the DSCs of LUL, LLL, RUL, RML, and RLL are 0.9579 ± 0.0125, 0.9479 ± 0.0157, 0.9507 ± 0.0133, 0.9003 ± 0.0331, and 0.9484 ± 0.0225, respectively. The PDV-Net model reported by Imran [15], select the chest CT data training model from the LIDC data set, the slice thickness of the scans ranged from 0.50 to 1.50 mm, and the in-plane resolution varied between 0.53 and 0.88 mm The DSC results of lung lobe segmentation of the model are 0.966 ± 0.014, 0.966 ± 0.037, 0.937 ± 0.031, 0.882 ± 0.057, and 0.956 ± 0.017. Park [16] adopts the 3D U-Net model and select chest CT scans of mild-to-moderate chronic obstructive pulmonary disorder (COPD) patients, Slice thickness ranged from 0.625 mm to 0.80 mm, the reported result is 0.9556 ± 0.013, 0.9701 ± 0.010, 0.9697 ± 0.007, 0.9306 ± 0.030, and 0.9697 ± 0.007. Compared with previous studies, although our image data slice thickness is up to 5 mm, our hybrid segmentation model achieves satisfactory results in clinical data sets with less information in the image and large boundary variation between successive layers.

Similar to that reported in previous studies, the DSC value of RML was lower than those of other lobes. The reasons may be as follows: First, in terms of volume, RML is much smaller than the upper and lower lobes, about 30–50% of the upper or lower lobe. Small errors in auto-segmentation volumes can lead to large differences in Dice value and reduce the overall performance. Second, segmentation errors generally occur at the starting slices, where the RML/RLL appears, especially for CT images with a thickness of 5 mm, since the fissures between RML and RUL or RLL are more difficult to recognize than those are displayed as lines on thin-section CT, the boundary of a middle or lower lobe in its starting slice is not often sensitively identified by segmentation network (Fig. 4), which makes accurate segmentation of RML a challenge. In the proposed hybrid network, the advantages of 2D and 3D networks are combined to improve the segmentation accuracy of RML. From our results, the mean DSC of RML reached 0.9, which was comparable to the results of other models in 1–2 mm slice thickness CT images. Among 175 contours, 11 contours required manual correction by the physician. We analyzed the cases that are significantly different from manual contours in the evaluation results. The main differences are in the continuous layers, where the contour of the lung lobes changes greatly in the superior–inferior direction, especially in the slices, where the fissures between adjacent lung lobes appear. However, most of the differences are considered acceptable for clinical use after visual assessment.

Our research has some limitations. Because our main research goal is to establish a hybrid model that can achieve automatic lung lobe segmentation on clinical standard CT images for locally advanced NSCLC patients undergoing radiotherapy and to evaluate the clinical applicability of the model. We did not perform much post-processing optimization to improve our segmentation results, which may also affect some of our segmentation results. We will further optimize our model in future work to achieve more precise lung lobe segmentation.

## Conclusion

The 2D–3D CNN hybrid segmentation model proposed in this study can achieve relatively accurate automatic segmentation of lung lobes on conventional chest CT of locally advanced lung cancer patients, and results that meet the needs of clinical applications can be obtained with minimal manual participation. Our model reduces the impact of heavy manual segmentation workload and time-consuming on clinical workflow and provides a basis for the implementation of lobar-level contouring, treatment planning, dose evaluation, and treatment outcome prediction for locally advanced lung cancer patients.

## Methods

### 2D–3D hybrid CNN

**Fig. 5:**
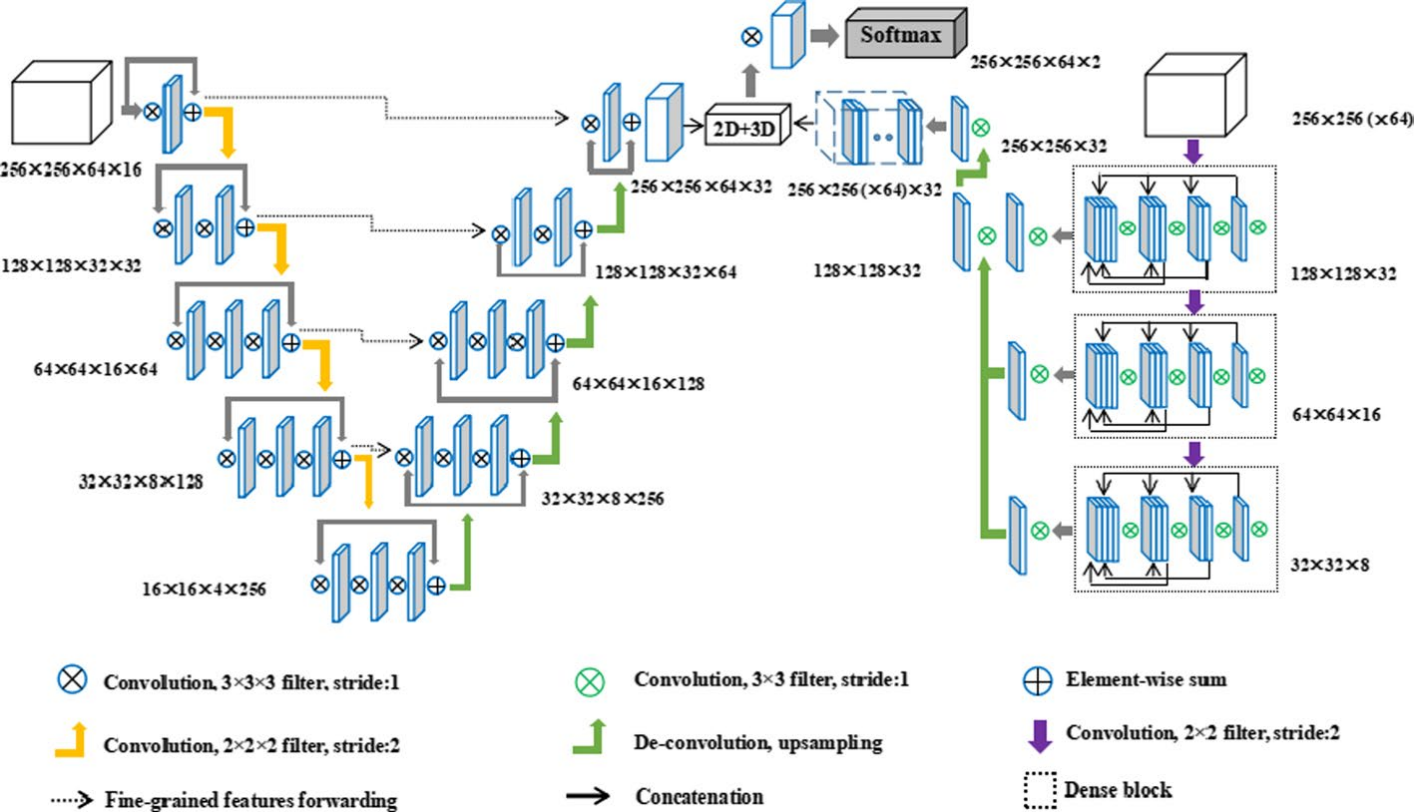
Schematic diagram of network structure

Our model is an end-to-end trainable hybrid convolutional neural network, which combines a 3D CNN to learn the long-range 3D contextual information of CT images, and a 2D CNN to capture intra-slice semantic information. The complete network structure diagram is shown in Fig. 5.

2D CNN adopts dense connection to ensure maximum information flow, which could capture more details in each layer for edge detection. The gradually decreasing number of channels in the encoding path increases the weight of the low-level information, which is beneficial to the network to focus on extracting the edge information.

2D features are effectively fused with 3D features through a hybrid features fusion module, which combines the complementary information of the two networks and integrates the information on different spatial scales. It solves the problem that 2D CNN cannot extract the volume information of CT image context, and alleviates the problem that 3D CNN is not sufficient in extracting intra-layer features. The Dice Loss function used by the network effectively alleviates the common problem of category imbalance in segmentation.

### 3D CNN

We employ the V-Net model proposed by Milletari [20] to extract the context information of lung lobes on CT images. The training volumes were first normalized, followed by rescaling to 512 × 512 × 64. The model is an encoder–decoder structure, where the encoder is used to extract features and reduce its resolution at the end of each stage, and the decoder is used to gradually restore the low-resolution features generated by the encoder to the same resolution as the input image through transpose convolution, the final output feature map of 3D CNN was 256 × 256 × 64 × 32, and the resolution is compressed for convolutional downsampling using a kernel size of 2 × 2 × 2 with stride 2. The resolution of the feature map is reduced by half in three directions (XYZ) with each down-sampling. The parametric rectified linear units (PReLU) activation function is used in the network.

### 2D CNN

The 2D CNN proposed in this paper has an encoding path similar to the V-net structure. Three dense blocks are set on the encoding path to extract features. Through the dense connection scheme, network feature propagation is promoted and feature utilization is enhanced so that 2D CNN can fully excavate the 2D texture information. We trained the network with axial slices from all the training volumes, each sized 256 × 256 and normalized to have values between 0 and 1. To avoid over-fitting to the background, only the axial slices, wherein at least one lung lobe is present were used. Different from V-net, the size of the feature map and the number of channels will be reduced after every down-sampling, which is beneficial for the network to concentrate on extracting the edge information of lung lobes on CT images. The decoding path eliminates most convolution processing for low-resolution feature maps, directly restores the resolution of feature maps through Transpose convolution processing, which improves the operating efficiency of the network.

### 2D–3D fusion

Before obtaining the final segmentation map, features extracted by 2D CNN and those extracted by 3D CNN need to be fused, and the feature maps of the two networks need to be consistent in size and dimension. The featured image extracted by 2D CNN becomes 256 × 256 × 32 after up-sampling, and the final output results of 64 consecutive CT slices in 2D CNN are combined to generate a tensor that can be used for 3D convolution. The size and channel number of the tensor are 256 × 256 × 64 × 32, which is the same as that of 3D CNN. After that, two-dimensional features and three-dimensional features are concatenated to form hybrid features. The mixed features are refined through a 3 × 3 × 3 convolution kernel and generate feature maps of two channels. Finally, a softmax layer is applied to generate the final segmentation.

Loss function: To capture the local and global relationship between different output pixel predictions of this hybrid network, the context information based on the Dice coefficient was used to correct the lobe shape information in both networks:

$$\mathop L\nolimits_{Dice} = 1 - \frac{{2\sum\nolimits_{i} {\mathop x\nolimits_{i} \mathop y\nolimits_{i} } }}{{\sum\nolimits_{i} {\mathop x\nolimits_{i}^{2} } + \sum\nolimits_{i} {\mathop y\nolimits_{i}^{2} } }},$$

where x_i_ is the prediction probability for each voxel and y_i_ is the binary ground truth.

### Data set

105 locally advanced non-small cell lung cancer patients with pathologically confirmed IIIA (N2) were retrospectively selected from the case database from June 2019 to August 2020. The diagnostic CT of the patient is in the supine position, lying on the ordinary CT diagnostic curved bed, and the arm is raised above the head. The reconstructed image has a resolution of 512 × 512 and a slice thickness of 5 mm. It is transmitted to MIM 7.0.4 (MIM vista Corp, Cleveland, US-OH). A total of 350 three-dimensional lung lobe contours, seventy contours per lobe was trained and validated after the image data augmentation process, so as to ensure the amount of data needed for model training. One experienced radiation oncologist contoured five lung lobes for each CT scan, and the data is saved in the RTstructure Dicom file.

### Implementation

Each CT slice is resampled at the same resolution (1 × 1 × 3 mm) and cropped to 256 × 256. For each CT sequence, a 64 × H × W 3D image was synthesized from 64 consecutive images with stride as 1. 105 patients were randomly divided into a training group (50 cases) to optimize the network, a validation group (20 cases) to determine the optimal performance model, and a test group (35 cases) to test and evaluate the model after complete training. The total data set includes 7090 CT slices, 4705 slices for the Training-Validation cohort, and 2385 slices for the Test cohort. The augmentation of input data was used to avoid overfitting and improve the generalization capabilities of deep neural networks. Random Translation, scaling, rotation, and other data enhancement techniques were used to increase the sample size.

The proposed CNN was trained on two NVIDIA 1080Ti GPU with 11 GB of RAM for 1000 epochs. The code was written in PyTorch Library using Python. Considering the GPU memory limitation, the batch size was set to 1. For the gradient descent optimization algorithms, we used the Adam optimizer with a learning rate of 0.001 and a weight decay of 10–8.

### Evaluation metrics

Multiple metrics were used to quantitatively evaluate the accuracy of the proposed segmentation method. The manual contours delineated by a single expert (RO) were considered ground truth in this study. Dice Similarity Coefficient (DSC) [21, 22] is the main evaluation metric that quantifies the spatial overlap between the ground truth and the automated contours, defined as

$${\text{Dice}} = \frac{{2{\text{|X}} \cap {\text{Y|}}}}{{{\text{|X|}} + {\text{|Y|}}}},$$

where X was the set of segmentation results and Y was the set of ground-truth delineation. The value of Dice varied from 0 to 1, and a higher value of Dice usually implied a better match between the two contours. A Dice score of 0.75 was considered an acceptable match in this study.

Hausdorff distance (HD) [22] is a measure of surface distance between two point sets, defined as

$${\text{HD}} = {\text{max}}\left\{ {\mathop {\max }\limits_{y \in Y} \mathop {\min }\limits_{x \in X} d(y,x),\mathop {\max }\limits_{x \in X} \mathop {\min }\limits_{y \in Y} d(x,y)} \right\},$$

where X and Y denoted the boundary-surface set of the automated contours and the ground truth, d (x, y) indicated the Euclidean distance between voxels x and y. 95th-percentile Hausdorff distance (HD95) describes the largest surface-to-surface separation among the 95th percentile of surface points of automated contours and ground truth. Hausdorff distance referred to the maximum distance of all surface voxels. However, it was sensitive to small outlying objects and HD95 was employed to skip the outliers. A smaller value of HD95 usually implied a better result.

Mean surface distance (MSD) [23] was defined as follows:

$${\text{MSD}} = \frac{{1}}{{2}}\left( {\frac{1}{{\left| {\text{Y}} \right|}}\sum\limits_{{{\text{y}} \in Y}} {\mathop {\min }\limits_{{{\text{x}} \in {\text{X}}}} d\left( {y,x} \right) + \frac{1}{\left| X \right|}\sum\limits_{{{\text{x}} \in X}} {\mathop {\min }\limits_{{{\text{y}} \in {\text{Y}}}} d\left( {x,y} \right)} } } \right).$$

Sensitivity, Specificity, and Accuracy [24] were also used as performance assessment parameters:

$$\begin{array}{*{20}c} {{\text{ Sensitivity }} = \frac{TP}{{TP + FN}}*100} \vspace{6pt}\\ {{\text{ Specificity }} = \frac{TN}{{FP + TN}}*100} \vspace{6pt}\\ {{\text{ Accuracy }} = \frac{TP + TN}{{TP + FN + TN + FP}}*100} \\ {} \\ \end{array}$$

where TP, TN, FP, and FN denotes true positive, true negative, false positive, and false negative correspondingly.

### Statistical analysis

All statistical comparisons were performed using SPSS software (version 22.0; IBM, Inc., Armonk, NY, USA). A value of P < 0.05 indicated statistical significance.

**Table 1:** Tumor location information of all cases

Location	Entire cohort	Training-validation cohort	Test cohort
LUL	32	22	10
LLL	12	9	3
RUL	22	16	6
RML	14	4	10
RLL	25	19	6
Total	105	70	35

*LUL* left upper lobe, *LLL* left lower lobe, *RUL* right upper lobe, *RML* right middle lobe, *RLL* right lower lobe.

**Table 2:** Quantitative parameters for lung lobe segmentation

N=35	LUL	LLL	RUL	RML	RLL
HD95 (mm)	22.3584±17.2096	20.9913±7.1894	16.9986±7.8134	26.553±13.995	23.4818±11.1656
MSD (mm)	0.9754±0.2355	1.2095±0.3613	1.1752±0.2935	1.9358±0.7122	1.2164±0.5285
DSC	0.9579±0.0125	0.9479±0.0157	0.9507±0.0133	0.9003±0.0331	0.9484±0.0225
Accuracy	99.5715±0.0928	99.5951±0.1209	99.6668±0.0892	99.7161±0.0707	99.5753±0.1421
Sensitivity	98.2261±0.6801	96.1441±1.5422	96.1279±1.7629	92.3785±3.6881	96.0335±2.0398
Specificity	99.6506±0.0652	99.7638±0.0603	99.8104±0.0756	99.8346±0.0762	99.7793±0.0638

*LUL* left upper lobe, *LLL* left lower lobe, *RUL* right upper lobe, *RML* right middle lobe, *RLL* right lower lobe.

## Data Availability

The data sets during and/or analysed during the current study available from the corresponding author on reasonable request.

## Abbreviations

2D, 3D: Two-dimensional, three-dimensional
CNN: Convolutional Neural Network
CT: Computed tomography
NSCLC: Non-small-cell lung cancer
DSC: Dice Similarity Coefficient
LUL: Left upper lobe
LLL: Left lower lobe
RUL: Right upper lobe
RML: Right middle lobe
RLL: Right lower lobe
RILI: Radiation-induced lung injury
HD: Hausdorff distance
HD95: 95Th-percentile Hausdorff distance
MSD: Mean surface distance
PVE: Partial volume effect
COPD: Chronic obstructive pulmonary disease
